# Abstract of Proceedings of the Buffalo Medical Association

**Published:** 1869-04

**Authors:** T. M. Johnson

**Affiliations:** Sec’y.


ART. IV—*Abstract of Proceedings of the Buffalo Medical Association.*

TUESDAY EVENING, April 6th, 1869.

The meeting was called to order by the President. Members present—Drs. Lothrop, Gay, Little, Miner, Samo, White, Wetmore, Ring, Brown, Phelps, Greene, Diehl and Johnson.

On motion the order of exercises was suspended to allow Dr. Weeks to exhibit a pessary.

The Secretary's Annual Report was read, accepted, and ordered placed on file.

The Treasurer's Annual Report was read, accepted, and referred to a committee of one. The chair appointed Dr. White such committee. Dr. White moved that the report be received. Carried.

DR. WHITE moved that a committee of three, including the Treasurer, be appointed to revise and correct the books, which are in an unsatisfactory condition. The motion was carried, and the chair appointed Drs. Samo and Wetmore to act with the Treasurer.

By vote, the Treasurer was authorized to procure blank receipts to be given members on payment of dues.

Election of officers for the ensuing year was then held, with the following result:

| | |
|---|---|
| President, . . . . . | J. F. MINER. |
| Vice President, . . . . . | S. W. WETMORE. |
| Secretary, . . . . . | T. M. JOHNSON. |
| Treasurer, . . . . . . | CONRAD DIEHL. |
| Librarian, . . . . . | J. B. SAMO. |

Dr. William O. Taylor was elected to membership.

DR. GREENE said: Mr. President and gentlemen, I have here a calculus or concretion, which to me is of much interest. The history of this concretion is this: it was dropped by a horse which belonged to myself, and found in the stable covered with manure. On being washed, it presented an oval form and greenish appearance, which became light brown on exposure to the atmosphere. It weighed twelve ounces, and when sawed through in the direction of its long diameter it was found to consist of layers of a crystaline substance, having a shingle nail as a nucleus. The length of this calculus in its long diameter is three and one-half inches; short diameter two and one-half inches. This horse, previous to this concretion being expelled, was subject to colic, but never since. I do not claim that this concretion has not a precedent, but I do believe they are very uncommon, from the fact that in examining a number of authors on the subject of intestinal concretion in the horse, only one mentions the existence of such. In Robert Jenning's second work on the horse, page 259, mention is made of a concretion weighing as high as six pounds. Through the kindness of Prof. George Hadley, we have chemical analysis, which I subjoin in full.

BUFFALO, April 5th, 1869.

Dr. Greene:—The intestinal concretion from a horse, submitted to me for examination, has a hardness slightly greater than gypsum. Heated before the blow-pipe it swells, darkens, gives off ammonia, and fuses with some difficulty into a white enamel. In muriatic or nitric acid it dissolves without effervescence, leaving a very little floculent organic matter. From this solution excess of ammonia throws down a precipitate, the whole of which is crystalline. Oxalate of ammonia gives no precipitate after careful neutralization. The nitric solution, on treatment with nitrate of silver and with ammonia, gives a brown yellow precipitate. Heated alone in a test tube the concretion gives off water. Heated with caustic potash solution, it evolves ammonia; and, after digestion, the portion remaining undissolved has with nitrate of cobalt the blow-pipe action of magnesia.

This concretion, therefore, contains no lime, very little organic matter, and consists almost entirely of phosphate of ammonia and magnesia. It is the triple phosphate of the books, (Mg. $NH^4PO^4$ $x^6H^2O$,) and is identical with the struvite of the mineralogists.

Yours, truly, GEORGE HADLEY.

DR. LITTLE reported the following case of a *post mortem* examination on a young person known as John R., aged 25 years, of Irish parentage, born in New York, of effeminate appearance, having no beard, and never having shaved his face. Had followed the occupation of cook on board of vessels on the lakes; belonged to a military company in the city, and served in the U. S. Army two years. *Post mortem* examination revealed no organs of generation, male or female; neither penis, scrotum or testicles, vulva, vagina, uterus or ovary, but what might be called a miniature penis, not as large as that of a new born male child. On examining it closely, outlines of labia could be seen.

The specimen was carefully removed for further investigation, by sawing through the pubes, and removing the parts in citu, with the bladder and part of the rectum and anus. Dr. Miner was present at the examination and perhaps has since, more carefully examined the parts, as he has the specimen in keeping for the museum of the Buffalo Medical College.

Dr. Miner said, that he had little to add to the description of Dr. Little. The subject had the general appearance and form of the female, and every one, judging from it alone, would unhesitatingly call him a woman; but upon examining the sexual region he would perhaps say, she is a man. Dr. Little has said that "examination revealed no organs of generation, male or female," and with his subsequent explanations he would endorse fully the statement. Certainly there are no developed generative organs natural to either man or woman. He had not himself, and he presumed Dr. Little had not made examination sufficient to be able to say that the neighboring parts contained no rudimentary organs answering to either sex. He had thus far avoided mutilating it by dissection, being desirous of preserving its natural appearance, but dissection could be made and still cause no injury to the specimen. The penis is rudimentary, but still has a foreskin, and as felt through foreskin, a glans penis. The bladder was greatly en larged, containing urine, which, on pressure, freely escaped through the urethra, which terminates naturally in the miniature penis. There are no testicles, unless they are to be found in the inguinal region or walls of the abdomen. There is no womb, unless it is rudimentary and entirely concealed in the tissues between the urethra and rectum; nothing like an ovary could be found in normal position. He thought it possible that careful dissection would reveal rudimentary organs which would more positively determine the sex. He mistrusted this might prove true, because it would be such a rare and perhaps unheard of anomaly, if no sexual apparatus could be found. If she was a man, she had many of the characteristics and appearances of a woman, and if he was a woman, certainly it must require most careful examination to demonstrate the presence of any of the female sexual organs. The case seemed remarkable to him, whatever might be shown by more careful examination.

Dr. White remarked that the case related by Drs. Miner and Little possessed great interest to the embryologist. The entire absence of genital organs, both male and female, was certainly very rare. In this instance, so far as could be discovered by the eminent gentlemen making the examination, there are no rudimentary organs of either sex. It is possible that upon a more careful inspection the rudimentary filaments may be discovered, having been arrested in their development at an early period in fœtal growth. Complete absence of the uterus has been doubted by many, because a more careful study of cases recorded as authority rendered the presence of rudimentary organs of one or the other sex presumable or evident. Simply examining the living is by no means conclusive on this point. To arrive at reliable conclusions a careful *post mortem* examination is important. A recent writer, Klob of Vienna, says "complete absence of the uterus, especially where accompanied with defective fallopian tubes and ovaries, is rarely found except in infants with an undeveloped condition of the lower half of the body and incapable of existence. Cases of absence of the uterus with complete development of the rest of the body have been but rarely met with."

Hermaphrodism or a commingling of the organs of the two sexes is much more frequently seen. Ordinarily when one of the male organs is absent a female of analogous character is substituted therefor. Complete hermaphrodism rarely if ever exists. It will be remembered by several members present that there is a cast in the museum of the University of Buffalo, presented me by my friend, the late Professor Ackley of Cleveland, which combines an unusually large proportion of the organs of both of the sexes. There is present the entire male organs, small in size and the testicles in the abdomen not having descended into the scrotum. There is also present a small uterus with fallopian tubes and ovaries. The lower end of the uterus is received into a small vagina which is connected with or rather opens into the male urethra. The external female organs only are wanting to complete the two sexes in one individual. The subject of this curious *lusus* had arrived at the age of twenty-four before he died, and was regarded as an effeminate *man*. He had had frequent sanguinolent discharges from the urethra, which during life, were supposed to come from the bladder or kidneys, but which are now believed to have been menstrual. He, like the individual described by Drs. Miner and Little, was not suspected of being mal-formed until it was disclosed in the dissecting room.

In a physiological point of view these cases deserve especial consideration. It will be remarked that the individual in whom all the organs were absent and the one in whom nearly all of both sexes were present, certainly all the most essential, were alike effeminate. It is well known that castration in the male produces effeminacy, whilst the loss of the ovaries has the effect of making the individual more masculine, the hair growing upon the face, the voice becoming harsh, and even the ossific pelvis becoming narrower, deeper, and in short, more like the male. A curious physiological inquiry as to what we are to expect of this *neuter*. Is *it* to resemble the female with extirpated ovaries, or the castrated male? But, Mr. President, I refrain from farther speculation until we may have this interesting specimen before us. I am happy to learn that the parts have been carefully preserved without mutilation, and will be submitted to the inspection of the members at no distant period. It certainly deserves the careful examination of all interested in this department, and it is highly probable that farther discussion will be elicited upon that occasion.

Dr. Lothrop had, through the kindness of Dr. Little, had an opportunity to examine the specimen removed from the body. Of the general appearances of the body as indicating sex he could say nothing, not having seen it; but such examination as he could make of the portion removed, without dissection, inclined him to disagree with the opinion that it belonged to a person of no sex. From his examination he was rather impressed with the belief that the person was a woman. In the specimen a large portion of the rectum was removed with the mass. The space, therefore, between the bladder and rectum had not been thoroughly explored. It seemed to him probable that careful dissection would disclose rudimentary female sexual organs. At any rate, until such dissection had been made, no positive conclusion as to sex could be adopted. The external organs appeared to him to have more resemblance to those of the female than to those of the male. The small, protruding organ, which was called a penis, he thought could have been formed by a prominence of the urethra or the union of the clitoris with it. He could not decide that there were any appearances like the gland penis or the prepuce. The prominences below the organ had quite as much the appearance of labia as of a scrotum. There was no opening to correspond to that of the vagina, but upon separating the slight folds the line of union had a pinkish tinge, and was not raised like the raphé of the scrotum. The arrangement of the hair seemed to him more like that usually seen upon the female. Whether right or not in these impressions, he was unwilling to admit that the person from whom these organs were removed, was of no sex. He was unwilling to believe in such a *lusus naturæ*, as an absolute absence of all trace of sexual organs, in a body otherwise well developed. He therefore desired farther examination in this case, as he could not now call to mind any recorded instance in which there was an entire absence of all organs indicative of sex, apart from other malformations, or defects of development.

Adjourned. T. M. JOHNSON, Sec'y.

